# Egg chemoattractants moderate intraspecific sperm competition

**DOI:** 10.1002/evl3.34

**Published:** 2017-11-28

**Authors:** Rowan A. Lymbery, W. Jason Kennington, Jonathan P. Evans

**Affiliations:** ^1^ School of Biological Sciences The University of Western Australia Crawley WA 6009 Australia

**Keywords:** Gamete interactions, genetic compatibility, sexual selection, sperm chemotaxis, sperm competition

## Abstract

Interactions among eggs and sperm are often assumed to generate intraspecific variation in reproductive fitness, but the specific gamete‐level mechanisms underlying competitive fertilization success remain elusive in most species. Sperm chemotaxis–the attraction of sperm by egg‐derived chemicals—is a ubiquitous form of gamete signaling, occurring throughout the animal and plant kingdoms. The chemical cues released by eggs are known to act at the interspecific level (e.g., facilitating species recognition), but recent studies have suggested that they could have roles at the intraspecific level by moderating sperm competition. Here, we exploit the experimental tractability of a broadcast spawning marine invertebrate to test this putative mechanism of gamete‐level sexual selection. We use a fluorescently labeled mitochondrial dye in mussels to track the real‐time success of sperm as they compete to fertilize eggs, and provide the first direct evidence in any species that competitive fertilization success is moderated by differential sperm chemotaxis. Furthermore, our data are consistent with the idea that egg chemoattractants selectively attract ejaculates from genetically compatible males, based on relationships inferred from both nuclear and mitochondrial genetic markers. These findings for a species that exhibits the ancestral reproductive strategy of broadcast spawning have important implications for the numerous species that also rely on egg chemoattractants to attract sperm, including humans, and have potentially important implications for our understanding of the evolutionary cascade of sexual selection.

**Impact Summary**

Gamete interactions are a critical component of competitive reproductive fitness. In many organisms, multiple mating (for internal fertilizers) or multi‐individual spawning (for external fertilizers) lead to competition among ejaculates for fertilization and the opportunity for females (or eggs) to promote the success of preferred sperm. However, despite the pervasiveness of these forms of sexual selection, we know very little about the specific mechanisms of interaction among eggs and sperm that underlie such processes. One emerging putative mechanism is sperm chemotaxis, a taxonomically widespread phenomenon involving the attraction of sperm toward eggs by egg‐derived chemicals. Here, we exploit the experimental versatility of a broadcast spawning mussel to provide the first empirical evidence that differential sperm chemotaxis allows females to bias the outcomes of intraspecific sperm competition toward sperm from “preferred” males. Additionally, patterns of genetic relatedness at both nuclear and microsatellite markers suggest that females base these chemoattractant‐induced preferences on complex patterns of genetic compatibility. Together, our results provide rare mechanistic insight into the interactions underlying gamete‐level sexual selection. Moreover, this mechanism (sperm chemotaxis) has the potential to play similar roles across many taxa, given the ubiquity of egg chemoattractants. Indeed, as broadcast spawning was the ancestral mode of reproduction, gamete‐level mechanisms that mediate competitive fertilizations likely played an important role in the evolution of sexual reproduction. The identification of such mechanisms, therefore, represents a crucial step forward in our understanding of sexual selection.

Sexual selection, which acts on variation in traits that influence reproductive success, almost certainly began in the sea with externally fertilizing organisms (Levitan 2010; Parker 2014). In these systems, before the evolution of advanced mobility and sensory structures, there would have been limited opportunity for mating competition or mate choice prior to gamete release. Instead, synchronous broadcast spawning (where gametes from both sexes are expelled externally) and the co‐occurrence of gametes from multiple individuals likely fuelled sexual selection in the form of sperm competition (competition for fertilization among ejaculates from multiple males; Parker 1970) and cryptic female choice (biasing of fertilization by females or their eggs toward particular ejaculates; Thornhill 1983; Eberhard 1996). Recent theory suggests that these ancestral processes of sexual selection instigated the evolutionary cascade toward many derived features of animal reproductive systems, including sexual dimorphism, internal fertilization, and precopulatory sexual selection (Parker 2014). However, sperm competition and cryptic female choice have themselves remained pervasive forms of sexual selection in most sexually reproducing taxa (Pitnick and Hosken 2010). There is, therefore, considerable empirical value in studying gamete‐level interactions in extant broadcast spawners as they may provide clues into the mechanisms underlying sperm‐egg interactions in a broad range of taxonomic groups (Levitan 2010; Evans and Sherman 2013).

A key goal in reproductive and evolutionary biology is to seek mechanistic insights into the processes that generate fertilization biases during sperm competition, and in particular into the role that females play in moderating this competition (Pitnick et al. 2009; Pitnick and Hosken 2010; Firman et al. 2017). While evidence for female control over fertilization is now compelling in many systems (e.g., Clark et al. 1999; Nilsson et al. 2003; Pilastro et al. 2004; Lovlie et al. 2013; Young et al. 2013; Firman and Simmons 2015), direct demonstrations of the underlying mechanisms remain largely elusive (but see Gasparini and Pilastro 2011; Alonzo et al. 2016). Broadcast spawning taxa offer particularly amenable and experimentally tractable systems with which to identify such mechanisms (Evans and Sherman 2013). Unlike internal fertilizers, in broadcast spawners the interactions between gametes are not hidden from view within the female reproductive tract, making it possible to visualize processes (e.g., gamete selection) that would otherwise have to be inferred indirectly. For example, eggs of broadcast spawners can moderate the recognition and fusion of sperm at the gamete surface (Palumbi 1999; Levitan and Ferrell 2006), or select specific sperm nuclei when multiple sperm penetrate the egg (Carré and Sardet 1984). However, eggs can also influence sperm remotely (i.e., prior to the meeting of gametes) through the release of chemical attractants. This process, which is known as sperm chemotaxis, is often crucial in broadcast spawners for ensuring eggs are found and fertilized by conspecific sperm (Miller et al. 1994; Riffell et al. 2004). Moreover, it has been argued that when ejaculates from multiple conspecific males are present, such remote signaling between eggs and sperm could be an important mediator of competitive fertilization success (Evans et al. 2012).

Although sperm chemotaxis is taxonomically widespread in both external and internal fertilizers (Miller 1985; Eisenbach 1999; Eisenbach and Giojalas 2006), its putative role in gamete‐level sexual selection has only recently come to light. For example, recent studies on the broadcast spawning mussel *Mytilus galloprovincialis* have revealed that chemoattractants have differential effects on the swimming behavior (chemotactic responses, swimming trajectory, and speed; Evans et al. 2012; Oliver and Evans 2014) and physiology (acrosome reaction; Kekäläinen and Evans 2016) of sperm from different conspecific males. The strength of these effects correlate with differences in offspring survival among male–female crosses (Oliver and Evans 2014). These findings suggest that chemoattractants could promote fertilizations by genetically compatible sperm, but this has yet to be investigated under conditions of sperm competition. Moreover, the molecular processes underlying potential genetic compatibility effects are unknown. For example, differential sperm chemotaxis may be driven by gamete‐level mechanisms that promote optimal levels of general offspring heterozygosity, which is often cited as an explanation of compatibility‐based gamete choice (Firman et al. 2017). Alternatively, more specific patterns of genetic compatibility may apply in *M. galloprovincialis* populations, which typically contain multiple mitochondrial DNA lineages as a result of historical migration patterns (Westfall and Gardner 2010; Dias et al. 2014). What is clear, however, is that the intraspecific effects of chemoattractants on fertilization have important fitness implications for both males and females in this system.

In this study, we test whether differential sperm chemotaxis moderates gamete‐level mate choice in *M. galloprovincialis*, and whether fertilization biases attributable to differential chemotactic responses reflect underlying patterns of genetic complementarity. Our experimental design allows us to measure competitive fertilization success directly, rather than the more usual method of estimating fertilization success indirectly from a male's paternity share. The latter method (paternity share) can be confounded by postfertilization effects on offspring viability that may not be related to sperm competitiveness (García‐González 2008a; García‐González and Evans 2011). Here, we overcome this problem using a fluorescent dye to label the mitochondria of sperm of competing males (Lymbery et al. 2016). In *M. galloprovincialis* and many other bivalves, embryos inherit both paternal and maternal mitochondria through a process termed doubly uniparental inheritance (DUI) (Zouros et al. 1994; Obata et al. 2006; Breton et al. 2007). In DUI, maternal mitochondria are inherited in the somatic tissue of all offspring, while the paternal mitochondria are ultimately transmitted to the germ line of male offspring (Breton et al. 2007). Initially, however, sperm mitochondria are transferred into all fertilized eggs (Obata et al. 2006). This feature of bivalve reproductive biology enables us to label sperm with a fluorescent mitochondrial vital dye and track their success during fertilization when labeled sperm from focal males compete with unlabeled rival ejaculates (Lymbery et al. 2016).

The primary aim of our study was to determine whether chemoattractants moderate competitive fertilization success in *M. galloprovincialis*. To test this we used a novel multistep experimental protocol involving multiple 2 × 2 factorial crosses to determine whether egg chemoattractants moderate the success of ejaculates when they compete to fertilize eggs (see Methods). We also tested whether fertilization biases induced by egg chemoattractants (ECs) reflect patterns of genetic complementarity between focal sperm competitors and female EC donors. Our highly controlled design enabled us to: (1) directly examine variation in competitive fertilization success using sperm dyes, therefore controlling for postfertilization effects on embryo viability; (2) separate the effects of males, females, and their interactions on competitive fertilization success; and (3) isolate the effect of differential chemical attraction as the female‐moderated mechanism for biasing competitive fertilizations. Importantly, our design controls for stochastic variation in fertilization that could be caused by random sampling of rival males, by using sperm from a standard rival to compete with the dyed sperm of focal males within each factorial (García‐González 2008b; García‐González and Evans 2011). Our ensuing results provide the first direct evidence in any system that differential attraction of sperm up an egg chemoattractant gradient moderates intraspecific competitive fertilization success. Furthermore, we find that fertilization biases induced by egg chemoattractants reflect both preferences for unrelated males at nuclear loci and the selection of the same mitochondrial DNA lineage, thus revealing the putative genetic benefits of gamete‐level mate choice in this system.

## Methods

### STUDY SPECIES AND SPAWNING

*Mytilus galloprovincialis* is a sessile, gonochoristic bivalve mollusc that forms large aggregations on intertidal substrates in temperate regions of both Hemispheres. *Mytilus galloprovincialis* is distributed across the southern coast of Australia (Westfall and Gardner 2010), with phylogenetic studies indicating that populations contain signatures of both a native Southern Hemisphere lineage and a more recent introduction of Northern Hemisphere individuals (Westfall and Gardner 2010; Colgan and Middelfart 2011; Dias et al. 2014). Nevertheless, there appears to have been extensive reproductive mixture of individuals from these different lineages in Australian populations (Westfall and Gardner 2013). We collected mussels from Woodman Point, Cockburn, Western Australia (32°14′ 03.6″S, 115°76′ 25″E) during the 2015 spawning season (June–September), and maintained them in aquaria of recirculating seawater at the University of Western Australia until required (within one week of collection). Spawning was induced using a temperature increase from ambient to 28°C (Lymbery et al. 2016). Once an individual began spawning and its sex was determined, we immediately removed it from the spawning tank, washed it in filtered seawater (FSW) to remove possible contaminating gametes, placed it in an individual 250 mL cup and covered it in FSW. Once gametes were suitably dense, we removed the spawning individuals, estimated egg concentration by counting the number of cells in a homogenized 5 μL sample under a dissecting microscope, and estimated sperm concentration from subsamples (fixed in 1% formalin) using an improved *Neubauer haemocytometer*. We used these estimates to dilute gametes to their required concentrations for ensuing trials (see below).

### EXPERIMENTAL OVERVIEW

**Figure 1:**
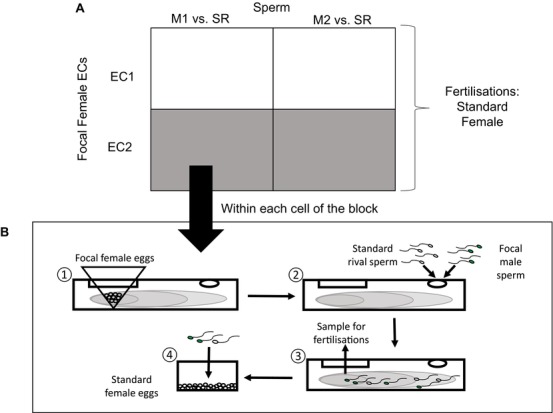
The overall design of an experimental block (A), and the steps performed within each cell of the block (B). (A) An example of one cross classified block, in which sperm from each of two focal males (M1 and M2) compete against sperm from a single standard rival (SR) in chemoattractant gradients from each of two focal females (F1 and F2). This generated four combinations per block, which were each replicated (*n* = 11 blocks, 44 combinations, 88 competitions total). Eggs from a single standard female per block were used to estimate competitive fertilization success. (B) The multistep competition assay illustrated using a single combination from within a block. (1) Eggs from the focal female were suspended in filter mesh to generate a chemoattractant gradient within the chamber. (2) The mesh and eggs were removed after 1 h, and dyed sperm from the focal male and undyed sperm from the standard rival added to the other end of the chamber. (3) After 10 minutes, a subsample was taken from the center of the chemoattractant gradient. (4) The subsample was added directly to eggs from the standard female, and competitive fertilization success of the focal male was measured.

We used a multistep cross‐classified design with blocks of two focal males (M1 and M2) and two focal females (F1 and F2) (Fig. 1A; the steps involved in a trial from a single cell of the block are shown in Fig. 1B). The initial steps involved differential sperm chemotaxis assays, where sperm from each focal male (dyed sperm, see below) competed with undyed sperm from a standard rival (SR) male in the presence of a chemoattractant gradient from each of the two focal females (EC1 and EC2). Therefore, four competitions were performed per block; M1 versus SR in EC1, M1 versus SR in EC2, M2 versus SR in EC1, and M2 versus SR in EC2. The final step involved competitive fertilization assays, where eggs from a single standard female (different to the focal females used for chemoattractant gradients) were used to assess the competitive fertilization success of the focal male (in competition with the standard rival) in each cross. This latter step enabled us to attribute differences in competitive fertilization success between competing ejaculates exclusively to the action of chemoattractant (i.e., it allows us to directly link differential chemotactic movement with the fitness outcome of sperm competition). Using eggs from a separate standard female for the fertilizations enables us to make this link by ensuring that within each block, the only source of male × female variation in competitive fertilization rates is through differential chemoattraction. The standard female eggs, which were the same throughout all cells of the block, would have had no confounding effect on male × female variation. We performed each competition in replicate, that is eight competitions per block (Fig. 1A), and conducted a total of 11 blocks (i.e., *n* = 22 focal males, 22 focal females, 44 male–female combinations, 88 competitions).

### COMPETITIVE CHEMOTAXIS AND FERTILIZATION TRIALS

In the first step of our experimental procedure, we established a chemoattractant gradient in an experimental chemotaxis chamber, then allowed dyed focal (M1 or M2) sperm and undyed rival (SR) sperm to swim in the chamber (Fig. 1B; these steps were performed for each cell of Fig. 1A). The chambers were made from sterile syringes (Terumo), with the ends of each syringe sawn off and sealed with parafilm (Bemis) to form a 10 mL tube. A ∼2 cm^2^ section was removed at one end of the chamber, and a small hole drilled in the other end. The chambers were fixed to a flat surface and a filter sack made of 30 μm filter mesh was inserted through the square opening. We added 5 mL of FSW to the chamber and 2 mL of egg solution (at 5 × 10^4^ cells mL^−1^) to the filter sack, which retained eggs but allowed chemoattractants to disperse into the chamber. We left the chambers for 1 h to establish a chemoattractant gradient (this time frame has previously been used to establish a chemoattractant gradient in larger chambers and we confirmed in preliminary trials that it was sufficient for our chambers; Evans et al. 2012).

Aliquots of sperm from the focal males and the standard rival were standardized to the same concentration (see below) and prepared for each competitive chemotaxis trial. The focal male's sperm was labeled using MitoTracker Green FM (Molecular Probes), prepared as described in Lymbery et al. (2016). In our previous study, we showed that dyeing sperm has no effect on sperm behavior or competitive ability (Lymbery et al. 2016). Apart from the addition of dye, focal male and standard rival sperm were treated to the same procedure. Briefly, 950 μL aliquots of sperm at 1 × 10^6^ cells mL^−1^ were prepared from each male, 50 μL of 500 nm dye solution added to focal male aliquots, and 50 μL of FSW added to rival aliquots. All samples (including undyed) were left in the dark (to prevent degradation of dye) for 10 minutes. The filter mesh containing focal female eggs was then removed from each chamber, and 500 μL each of focal male and standard rival sperm solution added to the drilled hole at the opposite end of the chamber (Fig. 1B). Sperm were allowed to swim in the gradient for 10 minutes. Preliminary trials confirmed that this assay did not result in any contamination of nonfocal sperm by excess dye from focal sperm (see Supplementary Methods).

After focal and rival sperm had been in the chemotaxis chamber for 10 minutes, 1 mL samples were taken from the center of the chemoattractant gradient (see Fig. 1B) and added to a separate petri dish containing 1 mL of FSW with eggs from the standard female (diluted to 1 × 10^4^ cells mL^−1^). Prior to the addition of sperm, we rinsed the standard eggs with FSW through 30 μm filter mesh to remove egg chemoattractants. However, even if these standard female eggs subsequently released chemoattractants, their impact (if any) would be to lessen our chance of detecting significant male‐by‐female effects (by obscuring patterns driven by the chemoattractants of focal females). Therefore, a significant male‐by‐female interaction in our analysis could only be attributable to the focal chemoattractants, which varied across the focal male samples. Moreover, fertilization occurs almost instantaneously upon the addition of sperm to the standard eggs (Lymbery et al. 2016), therefore decreasing the possibility that standard egg chemoattractants could reduce our power to detect effects. Although fertilization itself was instantaneous, we waited 10 minutes after the addition of sperm to allow dyed mitochondria to become visible inside fertilized eggs (Lymbery et al. 2016). We then estimated the fertilization success of the focal male under a fluorescent microscope by observing haphazard samples of 100 eggs, recording the numbers with and without dyed mitochondria.

Fertilizations from the rival (undyed) male were not scored, as estimating fertilizations from undyed sperm requires eggs to be left until they develop polar bodies, undergo cell division or until they can be assayed for survival. Therefore, the total numbers of fertilized eggs (dyed plus undyed) were not scored in this procedure. However, this is not required for the interpretation of the effects in our design, as we are not directly comparing the competitive success of focal males to rival males, but rather comparing the competitiveness of different focal males when they compete with a standard rival for standard eggs across different focal chemoattractants. Variation in the number of standard female eggs available for fertilization overall would only contribute to block‐level variation (as all trials within a block used eggs from the same standard female) and therefore would not systematically change the relative share of paternity among focal males within a block. Therefore, the male, female, and male x female effects (all nested within block) on competitive fertilization were not confounded by variation in proportion of standard female eggs available for fertilization.

### NUCLEAR GENETIC RELATEDNESS

Foot tissue samples from all focal males and focal females (i.e., egg chemoattractant donors) were preserved in 100% ethanol. DNA was extracted using a salt‐extraction method as described in Simmons et al. (2006) with the following alterations: tissue samples were incubated at 56°C overnight in the extraction buffer, and extracted DNA was resuspended in 100 μL of sterile water. DNA concentrations were estimated using a Nanodrop ND‐1000 spectrophotometer (Thermo Fisher Scientific) and DNA samples were stored at –20°C until required for PCR amplification. Each individual was genotyped at 13 polymorphic microsatellite loci; MGE002, MGE005, MGE008 (Yu and Li 2007), Mgu3 (Presa et al. 2002), Med744 (Lallias et al. 2009), MT282 (Gardestöm et al. 2008), MGES11 (Li et al. 2011), Mg‐USC20, Mg‐USC22, Mg‐USC25, Mg‐USC28, Mg‐USC42, and Mg‐USC43 (Pardo et al. 2011) (primer sequences provided in Table S1). Singleplex PCR reactions were run for each sample at each locus with a reaction volume of 5 μL, containing 1 μL MyTaq reaction buffer (Bioline), 0.2 μL primer mix (solution containing 10 nM each of forward and reverse primer, forward primer fluorescently labeled), 0.5 μL bovine serum albumin (Fisher Biotec), 0.1 μL MyTaq DNA Polymerase (Bioline), 2.2 μL sterile water, and 1 μL DNA sample (approximately 10 ng). PCRs were performed using an Eppendorf Mastercycler epGradient S, with an initial denaturation step at 95°C for 3 min, followed by 35 cycles of 95°C for 1 min, 54°C (MGE005 and MGE008) or 60°C (all other loci) for 1 min and 72°C for 1 min, with a final extension step of 72°C for 5 min. The PCR products were analyzed on an ABI 3730 96 capillary machine using a Genescan‐500 LIZ internal size standard, and genotypes for each locus were scored using GENEMARKER software (SoftGenetics). Peaks identified by GENEMARKER were checked manually and adjusted as necessary to minimize scoring errors.

One locus (MGES11) was monomorphic for our samples, with the number of alleles for the other 12 loci ranging from 3–20. We examined patterns of subpopulation variation and clustering of nuclear genotypes using the software program STRUCTURE (Pritchard et al. 2000, 2007; Falush et al. 2003; Supplementary Methods). Pairs of loci were tested for genetic linkage using likelihood ratio tests in GENEPOP (Raymond and Rousset 1995; Rousset 2008), with one pair of loci in significant linkage disequilibrium (Med744 and Mg‐USC22, *P* < 0.001). We therefore removed one of these loci from the analysis, specifically Med744 as there was also evidence of null alleles at this locus (Table S2; null alleles estimated using MICROCHECKER software; Van Oosterhout et al. 2004). There were excess homozygotes and evidence for null alleles at seven other loci (Table S2). However, removing all loci with null alleles can considerably reduce the power to detect variation in genetic relatedness and result in less accurate relatedness estimates than when all loci are included (Supplementary Methods; see also Robinson et al. 2013). We therefore used a maximum likelihood estimator that can account for null alleles (Kalinowski et al. 2006) to calculate genetic relatedness from the remaining 11 loci between each focal male–female pair in each block. These estimates were calculated using the ML‐RELATE software package (Kalinowski et al. 2006). We compared these estimates to a range of other relatedness estimators and found consistent patterns of variation in relatedness across different methods, increasing our confidence in the reported measures of nuclear genetic relatedness (see Supplementary Methods). Moreover, to determine whether any markers had a disproportionate effect on measures of relatedness, we examined whether relatedness changed when each marker was removed in turn, and found little variation across different combinations (Table S3).

### MITOCHONDRIAL HAPLOTYPES

We sequenced female‐type (F‐type) CO1 mtDNA, which is generally considered to have a more reliable phylogenetic signal than male‐type mtDNA and has multiple phylogenetic lineages in Australian *M. galloprovincialis* populations (Gérard et al. 2008; Colgan and Middelfart 2011; Dias et al. 2014). Using the DNA extracted as previously described, we amplified F‐type CO1 haplotypes using PCR reagents and conditions as described in Dias et al. (2014). Samples were sequenced in both directions by the Australian Genome Research Facility, Perth. Consensus sequences were aligned, analyzed and trimmed in Geneious v 6.1.8 (Kearse et al. 2012) using the Geneious alignment feature with default parameters. A preliminary Neighbor‐Joining tree was constructed from the 44 individuals to identify the number of unique sequences present (*n* = 14; Table S4). We added 105 northern and southern *Mytilus* haplotypes of the COI gene to our unique sequence set, as compiled in Dias et al. (2014). We inferred phylogenetic relationships using MRBAYES V3.1.2 (Huelsenbeck and Ronquist 2001) in Geneious v 6.1.8. We set the parameters and performed the Bayesian analyses as described in Dias et al. (2014), with the modification that we used a GTR+G substitution model. We determined phylogenetic relationships from 75% majority‐rule consensus of postburn‐in trees.

### STATISTICAL ANALYSES

Analyses were performed using R version 3.3.2 (R Core Team 2016). We first analyzed competitive fertilization success of focal sperm as a binomial response variable (proportion of eggs successfully fertilized by dyed sperm in competition). We fit a GLMM with logit link function in the “lme4” package (Bates et al. 2014), using the Laplace approximation of the log‐likelihood to estimate model parameters (Raudenbush et al. 2000). Our model included a fixed intercept term and random effects of male (overall variation among sperm of focal males), female (overall variation among focal female chemoattractants), male‐by‐female interaction (variation among sperm‐chemoattractant combinations), and experimental block. There was no overdispersion in our model (residual deviance = 77.15 on 83 degrees of freedom, dispersion parameter = 0.93), and the scaled residuals (calculated using the “DHARMa” package; Hartig 2017) were uniformly distributed (Kolmogorov–Smirnov test; D = 0.053, *P* = 0.967). Focal male competitive fertilization success ranged from 0% to 44%, that is significantly lower than 50% (fixed intercept term of GLMM = ‐1.79 [95% CIs = –2.11, –1.47], Wald Z = −1.78, *P* < 0.001). This was expected given only the subset of sperm that successfully traveled to the center of the chemoattractant gradient was used for fertilizations. We assessed the significance of random effect terms by removing each from the model in turn and compared the fit of the reduced models against the full model with likelihood ratio tests (–2 × difference in log likelihoods compared against χ^2^ distribution with 1 degree of freedom).

Next, we examined whether nuclear genetic relatedness and mitochondrial lineages of focal male and focal (i.e., chemoattractant‐producing) female pairs were predictive of competitive fertilization success. The replicate measures of competitive fertilization success for each combination of focal sperm and focal chemoattractant were significantly repeatable (*R* = 0.044 [95% CIs 0.023, 0.069], *P* < 0.001; estimated using GLMM method in the “rptR” package; Nakagawa and Schielzeth 2010). Therefore, the replicate measures were combined into weighted means (i.e., total fertilized out of total number of eggs across the two replicates). We fit a GLMM with logit link function to competitive fertilization success, with a continuous fixed effect of nuclear relatedness and a fixed categorical factor specifying whether the focal male and focal female pair had the same mitochondrial lineage or a different lineage. We also fit random effects of male, female, and block. There was no evidence of overdispersion in our model (residual deviance = 11.91 on 37 degrees of freedom, dispersion parameter = 0.32), nor heteroscedasticity of scaled residuals (Kolmogorov–Smirnov test; D = 0.079, *P* = 0.944). We used Wald Chi‐square tests to assess the significance of the fixed effects.

## Results

### COMPETITIVE FERTILIZATION SUCCESS

There were two sources of significant variation in focal male competitive fertilization success: (a) the male effect, and (b) the male‐by‐female interaction (Table 1). Although significant interactions often dictate that other effects must be interpreted cautiously, in this case the removal of both the male effect and the male‐by‐female interaction resulted in a significantly worse fit than removal of the male‐by‐female interaction alone (likelihood ratio statistic G^2^ = 68.80, *P* < 0.001). Therefore, the significant male effect suggests that there was variation among males in their average competitive success (i.e., some males were intrinsically “better” sperm competitors than others). The male‐by‐female interaction, on the other hand, indicates that there was significant variation in the way chemoattractants of focal females affected the competitive success of different focal males. In other words, the success of each focal male within a block depended on the specific identity of the focal female chemoattractant.

**Table 1:** Results of log‐likelihood ratio tests for random effects on focal male competitive fertilization success

Model	Log likelihood	AICc	G^2^	*P*
Full	−282.94	576.60		
(‐Male)	−285.89	580.26	5.90	0.015*
(‐Female)	−283.27	575.01	0.66	0.417
(‐Male × Female)	−285.41	579.30	4.95	0.026*
(‐Block)	−283.84	576.17	1.81	0.178

Full generalized linear‐mixed effects model included the proportion of eggs successfully fertilized by the focal male as the response variable (with logit link function), with random effects of focal male ID, focal female ID, male‐by‐female interaction and experimental block. The fixed intercept of the full model was significantly negative (intercept = –1.79 [95% CIs = –2.11, –1.47], Wald Z = –1.78, *P* < 0.001). Estimated variance components associated with random effects are provided in Table S5. Reduced models were fit by excluding each random effect in turn. Aikaike information criteria with correction for finite sample sizes (AICc) are provided for full and reduced models. The likelihood ratio statistic (G^2^) for each random effect was calculated as –2 × difference in log‐likelihoods between the relevant reduced model and the full model. Probability (*P*) statistics were estimated by comparing G^2^ to a χ^2^ distribution with one degree of freedom.

### GENETIC RELATIONSHIPS

The nuclear data indicated a well‐mixed population (Fig. S1), despite F‐type CO1 mtDNA haplotypes revealing signatures of two historical phylogenetic lineages (consistent with previously identified Northern and Southern Hemisphere lineages; Fig. S2; see also Dias et al. 2014). Nuclear genetic relatedness did not differ between focal male–female pairs that had the same mitochondrial lineage and those that had different mitochondrial lineages (two‐sample *t*‐test, *t*_42_ = 0.31, *P* = 0.759). We tested whether overall nuclear genetic relatedness or phylogenetic mtDNA lineages of focal male and focal (i.e., chemoattractant‐producing) female pairs predicted patterns of gamete‐level sexual selection (i.e., competitive fertilization success). We found significant main effects of both nuclear relatedness and mitochondrial lineage (Table 2). Specifically, competitive fertilization success was higher when focal male and focal female nuclear genotypes were less related, but also when focal males and focal females had the same mitochondrial lineage.

**Table 2:** Effects of nuclear genetic relatedness and phylogenetic mitochondrial lineage on competitive fertilization success

Fixed effect	Estimate	Χ^2^	*P*
Nuclear relatedness	−0.35 [–1.32, –0.02]	3.92	0.047
Mitochondrial lineage	0.35 [0.22, 0.65]	15.52	<0.001

Effects estimated from generalized linear‐mixed effects models of the proportion of eggs successfully fertilized by the focal male (with logit link function), with fixed effects of nuclear relatedness and mitochondrial lineage and random effects of focal male ID, focal female ID, and experimental block. The final model did not include the interaction term of the fixed effects, as the interaction was nonsignificant in the full model (Wald χ^2^ = 0.93, *P* = 0.335) and its inclusion reduced model fit (see Table S6; although significance of the main effects did not change with inclusion of the interaction). The fixed intercept of the model was significantly negative (intercept = –1.58 [95% CIs = –1.95, –1.22], Wald Z = –9.08, *P* < 0.001). Nuclear relatedness of focal male and focal female pairs was estimated from microsatellite loci using maximum likelihood (higher values = more closely related). Mitochondrial lineage (Northern or Southern Hemisphere) was assigned based on female‐type CO1 sequences, with focal male and focal female pairs scored as belonging to different or same lineage (estimate represents the mean change in fertilization success on the latent scale from different to same lineage). Hypothesis tests of main effects were conducted using Wald χ^2^ tests (d.f. = 1 for each effect).

## Discussion

Our results reveal that differential attraction of sperm up a chemical gradient can act as a mechanism of gamete‐level mate choice. To our knowledge, this is the first direct evidence that egg chemoattractants influence intraspecific sperm competition, supporting the previously documented differential effects of egg chemoattractants on sperm swimming direction (Evans et al. 2012), sperm motility (Oliver and Evans 2014), and sperm physiology (Kekäläinen and Evans 2016). We show that the effect of chemoattractants on competitive fertilization success depends upon the particular combination of focal male and focal female, specifically favoring certain genetic combinations over others. Previous work on this system has shown that the strength of sperm chemotactic responses for any given male–female pairing is positively correlated with offspring survival (Oliver and Evans 2014). These previous findings, together with the present results, suggest that egg chemoattractants allow females to promote fertilization by more compatible males when multiple ejaculates compete. This provides rare insight into the mechanisms used by females to gain control over the outcome of sperm competition.

Our results complement and extend recent evidence that female reproductive fluids more broadly can have important roles in gamete‐level sexual selection. In particular, there has been considerable interest in the ovarian fluid (OF) produced by various female fishes. In externally fertilizing salmonids, for example, OF released with eggs can differentially mediate the swimming speed of conspecific sperm depending on the particular male–female pairing (Urbach et al. 2005; Rosengrave et al. 2008; Butts et al. 2012). Although OF has yet to be implicated in intraspecific gamete‐level mate choice in salmonids (Evans et al. 2013), it has been shown to promote fertilization by conspecific sperm when in competition with those of sister species (Yeates et al. 2013). Intriguingly, however, there is evidence from an internally fertilizing poeciliid fish that OF within the female's reproductive tract can selectively bias fertilization in favor of sperm from unrelated males over related males (Gasparini and Pilastro 2011). Recent work on an externally fertilizing wrasse has also shown that OF can bias competitive fertilization success toward dominant “nest” males (i.e., directional cryptic female choice; Alonzo et al. 2016). Our findings for mussels complement these prior studies by showing that egg chemoattractants similarly play an important role in mediating intraspecific sperm competition, thus exposing a previously unforeseen mechanism of sexual selection that may occur more broadly in other taxa. We suggest that further investigation into the effects of female reproductive fluids, including egg chemoattractants, across a broader range of taxa will provide fruitful mechanistic insights into gamete‐level mate choice.

We also found that the competitive fertilization biases induced by egg chemoattractants reflect complex genetic relationships between the focal males and focal (i.e., chemoattractant producing) females. These results may shed some light on patterns of genetic compatibility that underlie competitive fertilization biases, given previous findings that differential chemotaxis is correlated with offspring fitness of male–female pairs (Oliver and Evans 2014). Competitive fertilization success was higher for focal males that had a lower overall genetic relatedness to focal females (based on neutral nuclear markers), which complements recent evidence in other taxa that preferences for genetically dissimilar males may drive compatibility‐based cryptic female choice (Gasparini and Pilastro 2011; Firman and Simmons 2015). Although we did not directly examine the extent of inbreeding in our population, homozygote excesses consistent with inbreeding are not uncommon in populations of broadcast spawners (Huang et al. 2000; Addison and Hart 2005; Kenchington et al. 2006), possibly due to the unpredictable patterns of spawning and recruitment in these systems (Hedgecock and Pudovkin 2011). Therefore, gamete‐level mechanisms of maximizing offspring heterozygosity may be important for individual reproductive fitness.

In contrast to the patterns of overall genetic relatedness, we also found a competitive fertilization bias toward males that had the same phylogenetic mitochondrial lineage as the female. Preferences based on phylogenetic lineage are not unexpected in Australian *M. galloprovincialis* populations, as Northern and Southern Hemisphere lineages had diverged in allopatry from the Pleistocene before the more recent introduction of Northern individuals (Hilbish et al. 2000; Gérard et al. 2008). Nevertheless, it appears that such preferences have not maintained reproductive isolation between lineages, with the admixture of nuclear genotypes in our population supporting previous findings for Australian populations (Westfall and Gardner 2013). Possibly, this could be due to lineage‐based patterns being offset by the preferences for less related nuclear genotypes. However, the precise fitness benefits of the mitochondrial lineage‐based biases deserve further investigation. For example, one possibility is that fertilization biases reflect cyto‐nuclear compatibilities brought about by the presence of divergent mitochondrial lineages; it would therefore be interesting to examine how preferences relate to nuclear genes involved in mitochondrial function. Moreover, we sequenced the female‐type mtDNA common to somatic tissues of both males and females, but the occurrence and transmission of male‐type mitochondria in sperm may further complicate patterns. Therefore, the precise genetic interactions between males and females that underlie chemoattractant‐driven fertilization biases in these systems remain to be fully resolved.

To provide further mechanistic insights into gamete‐level mate choice in this system we need to identify the chemical profiles of egg chemoattractants and determine how variation in these profiles correspond to patterns of differential sperm attraction. Chemoattractant molecules have not yet been identified in *M. galloprovincialis*, but several types of egg‐derived chemicals have been described in other broadcast spawners (reviewed in Evans and Sherman 2013). For example, in echinoderms, peptides released from eggs bind to guanylyl cyclase receptors on the sperm surface, triggering a signaling pathway that results in influxes of extracellular calcium ions and a corresponding flagellar beat pattern (Kaupp et al. 2006; Alvarez et al. 2014). However, to our knowledge there has been no examination of intraspecific variation in such signaling pathways in any species. Recent evidence suggests that sperm‐activating peptides are evolutionarily conserved and vary little within genera (Jagadeeshan et al. 2015). Therefore, it may be unlikely that a single molecule type (such as a particular peptide) is responsible for intraspecific variation in sperm chemoattraction. Instead, it is possible that eggs release a variety of molecules that affect such signaling pathways. Our finding that the interacting effects of parental genotypes drive chemoattractant preferences suggests that these chemical signals are likely to be complex. Clearly there is a need to characterize intraspecific variation in egg chemoattractant chemical profiles to address these questions.

In conclusion, we provide the first direct evidence that egg chemoattractants moderate sperm competition and complement these findings with genetic data that may explain the previously documented offspring fitness benefits associated with differential sperm chemotaxis (Oliver and Evans 2014). Given our focus on a species exhibiting the ancestral mating strategy of broadcast spawning, and the fact that egg chemoattractants are found throughout a diverse range of taxa (Miller 1985; Eisenbach 1999; Teves et al. 2009), we anticipate that such mechanisms of gamete‐level mate choice may be prevalent in other species. However, until now the putative role of sperm chemotaxis in mediating intraspecific sperm competition has been largely untested. This is likely due in part to the empirical difficulty of linking the effect of putative mechanisms of gamete‐level mate choice directly to variation in competitive fertilization success. We demonstrate that powerful and tightly controlled experimental designs can provide detailed insights into the intricacies of gamete‐level sexual selection.

Associate Editor: R. Snook

## Supporting information


**Table S1**. Primer sequences, size range (Bp, base pairs) expected from literature (observed size range in parentheses) and references for the 13 microsatellite loci for *M. galloprovincialis* used in this study.
**Table S2**. Tests for null alleles at 13 microsatellite loci for *Mytilus galloprovincialis*, performed with Bonferroni correction for multiple tests using MICROCHECKER.
**Table S3**. Mean and standard error of maximum likelihood genetic relatedness of focal male – focal female pairs, estimated from all 11 microsatellite markers used in the final analysis and combinations with each marker removed in turn.
**Table S4**. F‐type CO1 haplotypes of *Mytilus galloprovincialis* recorded in our study.
**Table S5**. Link‐scale approximation of variance components associated with random effects in the full generalized linear mixed model of competitive fertilization success.
**Table S6**. Comparison of models with different combinations of the fixed effects nuclear genetic relatedness, mitochondrial lineage and their interaction on competitive fertilization success.
**Figure S1**. Bayesian modelling of subpopulation structure in microsatellite data, comparing the probability of models with different numbers of clusters (K = 1–5).
**Figure S2**. Bayesian phylogentic tree for *Mytilus* spp. female‐type CO1 mitochondrial DNA haplotypes, rooted in *M. trossulus* haplotypes.
Supplementary Methods: Sperm dye contamination trials; Testing for subpopulation structure of nuclear genotypes; Comparing performance of genetic relatedness measures.
