# Supplementary material for: Egg chemoattractants moderate intraspecific sperm competition
Source: Evol Lett. 2017 Nov 28;1(6):317–27. doi: 10.1002/evl3.34 (PMC6121861; doi:10.1002/evl3.34)
Supplement: Supplementary file 1 — Table S1. Primer sequences, size range (Bp, base pairs) expected from literature (observed size range in parentheses) and references for the 13 microsatellite loci for M. galloprovincialis used in this study. Table S2. Tests for null alleles at 13 microsatellite loci for Mytilus galloprovincialis, performed with Bonferroni correction for multiple tests using MICROCHECKER. Table S3. Mean and standard error of maximum likelihood genetic relatedness of focal male – focal female pairs, estimated from all 11 microsatellite markers used in the final analysis and combinations with each marker removed in turn. Table S4. F‐type CO1 haplotypes of Mytilus galloprovincialis recorded in our study. Table S5. Link‐scale approximation of variance components associated with random effects in the full generalized linear mixed model of competitive fertilization success. Table S6. Comparison of models with different combinations of the fixed effects nuclear genetic relatedness, mitochondrial lineage and their interaction on competitive fertilization success. Figure S1. Bayesian modelling of subpopulation structure in microsatellite data, comparing the probability of models with different numbers of clusters (K = 1–5). Figure S2. Bayesian phylogentic tree for Mytilus spp. female‐type CO1 mitochondrial DNA haplotypes, rooted in M. trossulus haplotypes. Supplementary Methods: Sperm dye contamination trials; Testing for subpopulation structure of nuclear genotypes; Comparing performance of genetic relatedness measures. [file EVL3-1-317-s001.docx]

**SUPPLEMENTARY MATERIALS**

**Egg chemoattractants moderate intraspecific sperm competition**

Rowan A. Lymbery, W. Jason Kennington, Jonathan P. Evans

**Supplementary Methods**

*Sperm dye contamination trials*

We conducted preliminary trials to determine whether adding dyed focal sperm solution and undyed rival sperm solution to the chemotaxis chamber would result in any contamination of rival sperm cells with dye. In these trials, we prepared dyed and undyed sperm samples following the same procedure as described in the main text. We then centrifuged the dyed sperm sample and collected the supernatant; i.e. the part of the dyed sample that would contain any excess dye solution but not the sperm cells themselves. This supernatant was then added to 7 mL of filtered seawater (i.e. replicating the volume of the chemotaxis chambers) along with the prepared undyed sperm sample. After 10 minutes (i.e. the length of time sperm were allowed to swim in the chemotaxis chamber in the experiment), we took subsamples from this solution and checked them under a Zeiss Axio Imager A1 fluorescent microscope. In haphazard counts of 100 sperm cells identified under normal light microscope, we did not find any contaminated by dye (n = 5 trials).

*Testing for subpopulation structure of nuclear genotypes*

We tested for subpopulation structure by analysing the microsatellite data with the software programme STRUCTURE (Pritchard et al. 2000, 2007; Falush et al. 2003). This programme uses Bayesian clustering analysis to assign individuals to K genetically distinct clusters. We did not use prior information about the location of samples (as all individuals were sampled from the same location). Our model parameters assumed admixture and correlated allele frequencies. We conducted 10 independent runs each for values of K ranging from 1-5, each with a burn-in of 10,000 followed by 100,000 Markov chain Monte Carlo (MCMC) iterations. We determined the most likely number of clusters by comparing the log probability of models with different values of K and the rate of change in log probability between successive values of K (ΔK) (Evanno et al. 2005).

*Comparing performance of genetic relatedness measures*

To check that our observed patterns of genetic relatedness were not an artefact of the relatedness estimator that we report in the main text (a maximum likelihood estimator that can account for null alleles, here denoted as ML-r), we compared a range of other relatedness measures for our loci. We first compared the performance of five different moment estimates of relatedness using the ‘compareestimators’ function of the ‘related’ R package (Pew et al. 2015). Briefly, the program uses observed genotype data to simulate genotypes from individuals of set levels of relatedness (full-sib, half-sib, parent-offspring and unrelated), and then compares the pairwise estimates of relatedness for these simulated individuals to theoretical expectations (0.5 for full-sib and parent-offspring, 0.25 for half-sib, 0 for unrelated). The correlation between the simulated relatedness estimates and the theoretical expectations can be used to assess the performance of each particular estimator for the observed loci. We found that all five moment estimators correlated strongly with theoretical expectations (*r* ranged from 0.70 - 0.76 for the five estimators), with the estimators of Lynch and Ritland (1999) (here denoted LR; *r* = 0.76) and Queller and Goodnight (1989) (QG, *r* = 0.71) best matching theoretical expectations. Note that if we included only observed genotypes at loci that did not show evidence of null alleles (see Supplementary Table 2) in the analysis, then the relatedness estimators performed more poorly against theoretical expectations (correlation *r* between estimators and theoretical expectations ranged from 0.49 - 0.52). This validates our expectation that removing these loci would reduce the power to detect variation in genetic relatedness and decrease the accuracy of estimators (Robinson et al. 2013).

The ‘related’ program can also determine the performance of a further two maximum likelihood methods of calculating relatedness, a dyadic likelihood method (Milligan 2003) and a triadic likelihood method (Wang 2007). However, these are computationally intensive to simulate and can only be compared to other estimators using manual code. We therefore compared only the best two moment estimators (as described above) to the two maximum likelihood estimators using manual code provided by Frasier (Frasier 2015). We found that the maximum likelihood estimators performed similarly to the moment estimators (*r* ranged from 0.70 - 0.72, note these are slightly different from the previous correlation coefficients when using the moment estimators only due to the random nature of simulations). We therefore used the best maximum likelihood estimator (triadic estimator, Tri) and the best two moment estimators (LR and QG) in further comparisons.

We determined whether the pairwise relatedness estimates provided by LR, QG and Tri were similar to pairwise relatedness calculated by ML-r (as reported in the main text). We used mantel tests to compare pairwise genetic relatedness matrices between the methods, and found that all three alternative methods from the ‘related’ package correlated strongly with the ML-r estimates (LR: *r* = 0.78, *p* < 0.001; QG: *r* = 0.71, *p* < 0.001; Tri: *r* = 0.85, *p* < 0.001). The consistent patterns across various relatedness estimators, together with the concordance with theoretical expectations in simulations, increases our confidence in the results we obtained using the ML-r estimator that accounts for null alleles (as presented in the main text).

**Supplementary Tables**

**Table S1.** Primer sequences, size range (Bp, base pairs) expected from literature (observed size range in parentheses) and references for the 13 microsatellite loci for *M. galloprovincialis* used in this study.

| Locus | Primer sequence | Bp range | Reference |
| --- | --- | --- | --- |
| MGE002 | **F**: GGTAGTTGGAGTGGTTGGT  **R**: AATGGTCGGTAGATGAAAAT | 272-280 (269-290) | (Yu and Li 2007) |
| MGE005 | **F**: CGTTGCCATCGTTTATTTT  **R**: GTTGTAAGTCGTGTTGGTTCA | 220-244 (226-247) | (Yu and Li 2007) |
| MGE008 | **F**: TGCTAAAAGTAATAAGACAGAT  **R**: GAGACCTCCAATAAATAAAA | 268-286 (272-280) | (Yu and Li 2007) |
| Mgu3 | **F**: AAACTAAAAACTTCATCTAATCCC  **R**: AAGCAATCCAAAGTGAGAGG | 143-151 (134-148) | (Presa et al. 2002) |
| Med744 | **F**: TTTTTCATCGTGTTTTGGTTG  **R**: CGCCATGGAATAGCCAATAG | 220-296 (188-231) | (Lallias et al. 2009) |
| MT282 | **F**: TGCCACATTGTTTTCAAGGA  **R**: TTCACGACAGCGACTATGAAA | 336-354 (332-350) | (Gardestöm et al. 2008) |
| MGES11 | **F**: CATCCCCGTATGGACATCAAG  **R**: ATCTGACACTGTGCAAATTGAGATC | 191-254 (180-180) | (Li et al. 2011) |
| Mg-USC20 | **F**: TACAGAAACGCCATGTCAGC  **R**: TGAGGTTCAAAGAACGGAAGA | 284-308 (284-318) | (Pardo et al. 2011) |
| Mg-USC22 | **F**: CCACAGGCACAACAAGTGTC  **R**: GCCACGTCTATAATGGCAGAA | 284-308 (380-384) | (Pardo et al. 2011) |
| Mg-USC25 | **F**: TCAGGTATCGTGACGGAAAT  **R**: GCAATAGATGCGCCTTCTTC | 272-308 (251-276) | (Pardo et al. 2011) |
| Mg-USC28 | **F**: CCACTGGACGTTAAGCAACC  **R**: CAAGCTCAATAGCTGGCTGA | 169-181 (161-169) | (Pardo et al. 2011) |
| Mg-USC42 | **F**: CTACCGGGCCTCATTTATCA  **R**: GCATCGTATTACCGGAGCAT | 138-146 (130-136) | (Pardo et al. 2011) |
| Mg-USC43 | **F**: TCCAAGATGGTTTAGCATTGG  **R**: TGGTGTATCCCTCCATGACT | 207-217 (199-212) | (Pardo et al. 2011) |

Polymerase chain reaction conditions are reported in the main text.

**Table S2.** Tests for null alleles at 13 microsatellite loci for *Mytilus galloprovincialis*, performed with Bonferroni correction for multiple tests using MICROCHECKER (Van Oosterhout et al. 2004).

| Locus | Observed homozygotes | Expected homozygotes | Null alleles present | Estimated null allele frequency |
| --- | --- | --- | --- | --- |
| MGE002 | 37 | 32.11 | Yes | 0.18 |
| MGE005 | 23 | 8.96 | Yes | 0.21 |
| MGE008 | 31 | 17.77 | Yes | 0.25 |
| Mgu3 | 24 | 21.24 | No | NA |
| Med744 | 18 | 8.80 | Yes | 0.15 |
| MT282 | 27 | 9.33 | Yes | 0.26 |
| MGES11 | 44 | 44 | NA | NA |
| Mg-USC20 | 20 | 19.77 | No | NA |
| Mg-USC22 | 21 | 21.25 | No | NA |
| Mg-USC25 | 30 | 23.70 | Yes | 0.20 |
| Mg-USC28 | 35 | 33.10 | Yes | 0.14 |
| Mg-USC42 | 18 | 18.33 | No | NA |
| Mg-USC43 | 21 | 12.41 | Yes | 0.15 |

Numbers of observed and expected homozygotes are reported, as well as estimated null allele frequencies if present. Note that locus MGES11 was monomorphic and provided no information for relatedness analyses, while locus Med744 was not included in relatedness analyses as it was in linkage disequilibrium with locus Mg-USC22 (see main text).

**Table S3.** Mean and standard error of maximum likelihood genetic relatedness of focal male – focal female pairs, estimated from all 11 microsatellite markers used in the final analysis and combinations with each marker removed in turn.

| Marker combination | Mean relatedness | SE relatedness |
| --- | --- | --- |
| All | 0.125 | 0.024 |
| MGE002 removed | 0.136 | 0.025 |
| MGE005 removed | 0.132 | 0.027 |
| MGE008 removed | 0.119 | 0.024 |
| Mgu3 removed | 0.127 | 0.028 |
| MT282 removed | 0.127 | 0.024 |
| Mg-USC20 removed | 0.120 | 0.023 |
| Mg-USC22 removed | 0.132 | 0.027 |
| Mg-USC25 removed | 0.138 | 0.026 |
| Mg-USC28 removed | 0.121 | 0.024 |
| Mg-USC42 removed | 0.134 | 0.026 |
| Mg-USC43 removed | 0.123 | 0.025 |

**Table S4.** F-type CO1 haplotypes of *Mytilus galloprovincialis* recorded in our study.

| Haplotype | Number of individuals | Previously recorded | Lineage | GenBank accession number |
| --- | --- | --- | --- | --- |
| H1 | 2 | Haplo7 | Northern | KF705219 |
| H2 | 8 | Haplo8 | Northern | KF705220 |
| H3 | 3 | Haplo4 | Northern | KF705216 |
| H4 | 15 | Haplo1 | Northern | KF705213 |
| H5 | 1 | Haplo9 | Northern | KF705221 |
| H6 | 2 | Haplo5 | Southern | KF705217 |
| H7 | 1 | Haplo6 | Southern | KF705218 |
| H8 | 2 | NA | Southern | MF462182 |
| H9 | 5 | Haplo3 | Southern | KF705215 |
| H10 | 1 | NA | Southern | MF462183 |
| H11 | 1 | NA | Southern | MF462184 |
| H12 | 1 | NA | Southern | MF462185 |
| H13 | 1 | Haplo14 | Southern | KF705226 |
| H14 | 1 | Haplo12 | Southern | KF705224 |

Number of individuals per haplotype are reported, along with previously recorded haplotype IDs if available (as per Dias et al. (2014), haplotype lineage and GenBank accession numbers (for both new and previously recorded haplotypes).

**Table S5.** Link-scale approximation of variance components associated with random effects in the full generalized linear mixed model of competitive fertilization success.

| Random effect | Variance component estimate |
| --- | --- |
| Male | 0.144 |
| Female | 0.028 |
| Male x Female | 0.051 |
| Block | 0.131 |
| Residual* | 7.525 |

Full generalised linear mixed-effects model included the proportion of eggs successfully fertilised by the focal male as the response variable (with logit link function), with random effects of focal male ID, focal female ID, male-by-female interaction and experimental block. The significance of random effects was tested with log-likelihood ratio tests (see main text).
*The link-scale approximation of the residual variance was estimated using the ‘rptR’ package (see description of method in Nakagawa and Schielzeth 2010; Schielzeth and Nakagawa 2013).

**Table S6.** Comparison of models with different combinations of the fixed effects nuclear genetic relatedness, mitochondrial lineage and their interaction on competitive fertilization success.

| Fixed effects | d.f. | ΔAICc | Weight |
| --- | --- | --- | --- |
| Relatedness, Lineage, Relatedness x Lineage interaction | 37 | 1.74 | 0.203 |
| Relatedness, Lineage | 38 | - | 0.535 |
| Relatedness | 39 | 3.56 | 0.001 |
| Lineage | 39 | 1.43 | 0.261 |

Residual degrees of freedom, difference in corrected Aikaike Information Criterion (AICc) between each model AIC and the minimum AIC (model including nuclear relatedness and mitochondrial lineage), and Akaike weights are presented for each model. The best-supported model included nuclear relatedness and mitochondrial lineage, but no interaction. Note that both nuclear relatedness and mitochondrial lineage had a significant effect on competitive fertilization success (Wald χ^2^ tests; see main text), regardless of whether the interaction term was included.


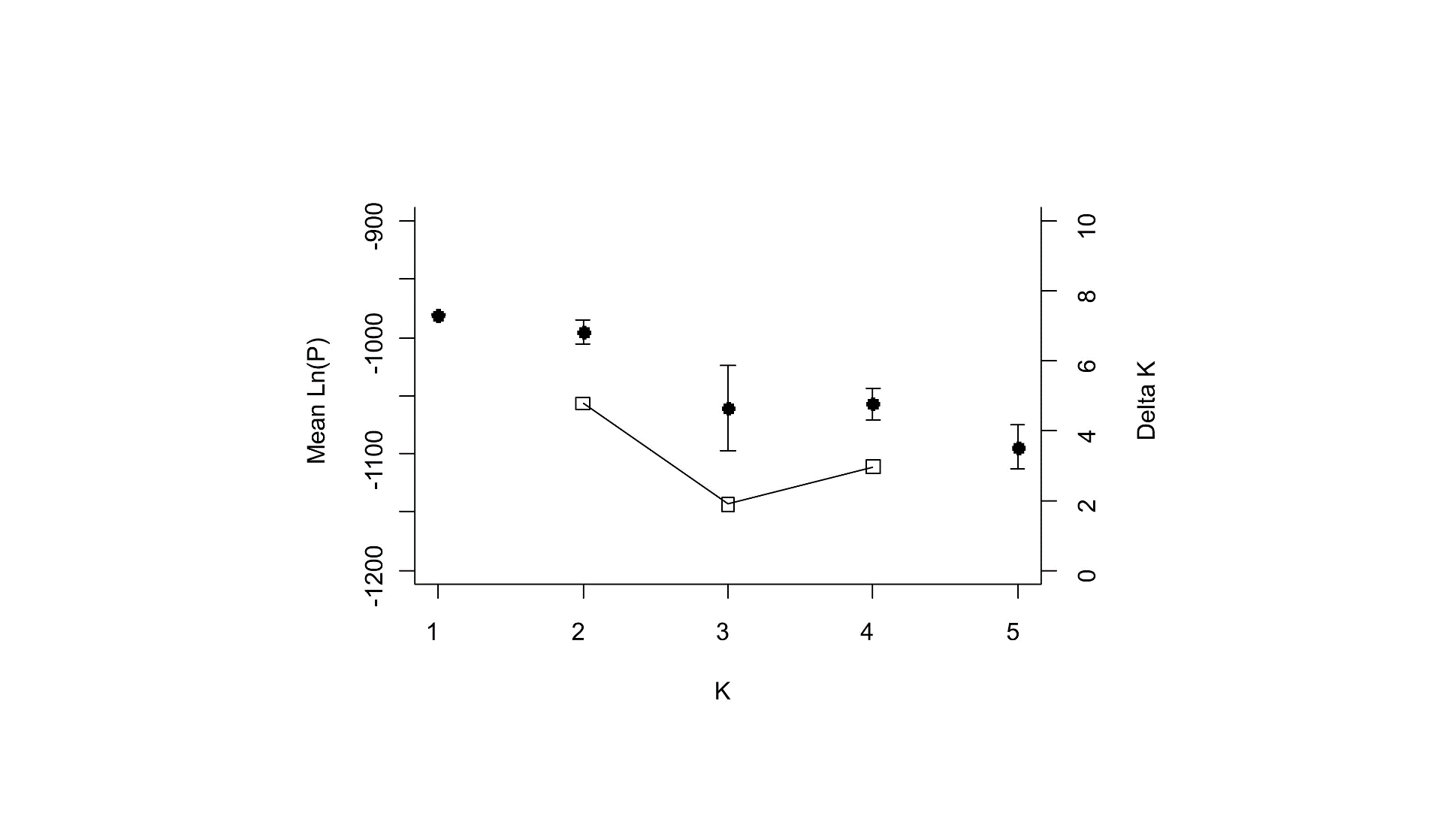
**Supplementary Figures**

**Figure S1.** Bayesian modelling of subpopulation structure in microsatellite data, comparing the probability of models with different numbers of clusters (K = 1-5). Points represent estimated mean (± standard deviation) log probability (solid circles) of models for each specified K, and rate of change in log probability between successive values of K (Delta K, open squares). The best supported number of clusters was K = 1; log-probability of models decreased as the number of hypothesised genetic clusters (K) increased. Moreover, in models with K > 1 most individuals were admixed, which is expected in the absence of real subpopulation structure (Pritchard et al. 2007).


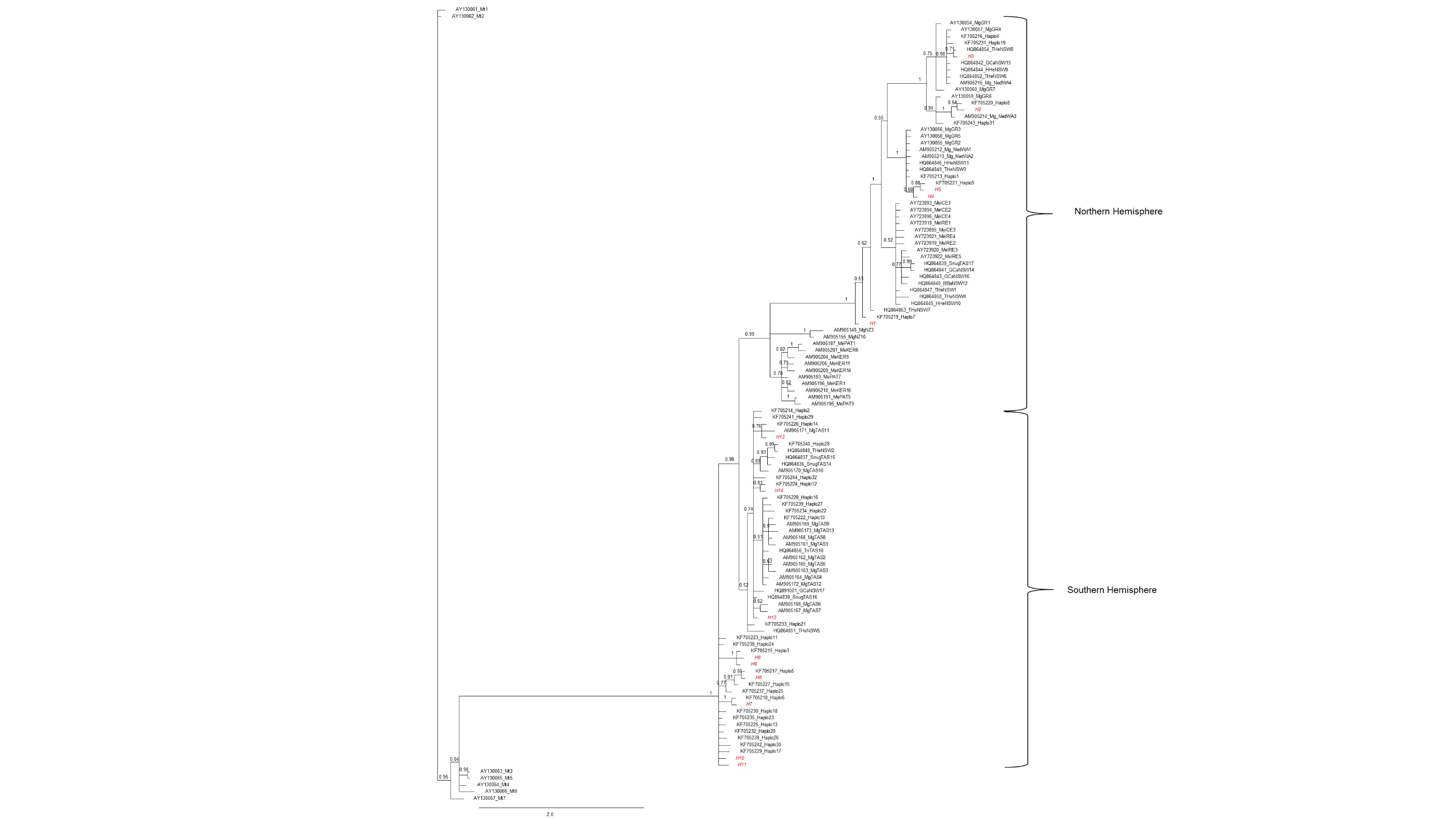


**Figure S2.** Bayesian phylogentic tree for *Mytilus* spp. female-type CO1 mitochondrial DNA haplotypes, rooted in *M. trossulus* haplotypes. The 14 unique haplotypes from our study (H1-H14) are shown in red and italics. Other haplotypes (105) were added from those compiled in Dias et al. (Dias et al. 2014); accession numbers for these sequences are provided in the names. Northern and Southern Hemispherelineages are identified on the right hand side.

**Supplementary References**

Dias, P. J., S. Fotedar, and M. Snow. 2014. Characterisation of mussel (*Mytilus* sp.) populations in Western Australia and evaluation of potential genetic impacts of mussel spat translocation from interstate. Mar. Freshw. Res. 65:486–496.

Evanno, G., S. Regnaut, and J. Goudet. 2005. Detecting the number of clusters of individuals using the software STRUCTURE: A simulation study. Mol. Ecol. 14:2611–2620.

Falush, D., M. Stephens, and J. K. Pritchard. 2003. Inference of population structure using multilocus genotype data: linked loci and correlated allele frequencies. Genetics 164:1567–1587.

Frasier, T. R. 2015. Tutorial For Using related.

Gardestöm, J., R. T. Pereyra, and C. André. 2008. Characterization of six microsatellite loci in the Baltic blue mussel *Mytilus trossulus* and cross-species amplification in North Sea *Mytilus edulis*. Conserv. Genet. 9:1003–1005.

Lallias, D., R. Stockdale, P. Boudry, S. Lapegue, and A. R. Beaumont. 2009. Characterization of 10 microsatellite loci in the blue mussel Mytilus edulis. J. Shellfish Res. 28:547–551.

Li, H. J., Y. Liang, L. J. Sui, X. G. Gao, and C. B. He. 2011. Characterization of 10 polymorphic microsatellite markers for Mediterranean blue mussel *Mytilus galloprovincialis* by EST database mining and cross-species amplification. J. Genet. 90:E30–E33.

Lynch, M., and K. Ritland. 1999. Estimation of pairwise relatedness with molecular markers. Genetics 152:1753–1766.

Milligan, B. G. 2003. Maximum-likelihood estimation of relatedness. Genetics 163:1153–1167.

Nakagawa, S., and H. Schielzeth. 2010. Repeatability for Gaussian and non-Gaussian data: a practical guide for biologists. Biol. Rev. 85:935–956.

Pardo, B. G., M. Vera, A. Pino-Querido, J. A. Álvarez-Dios, and P. Martinez. 2011. Development of microsatellite loci in the Mediterranean mussel *Mytilus galloprovincialis*. Mol. Ecol. Resour. 11:586–589.

Pew, J., P. H. Muir, J. Wang, and T. R. Frasier. 2015. related: An R package for analysing pairwise relatedness from codominant molecular markers. Mol. Ecol. Resour. 15:557–561.

Presa, P., M. Perez, and A. P. Diz. 2002. Polymorphic microsatellite markers for blue mussels (*Mytilus* spp.). Conserv. Genet. 3:441–443.

Pritchard, J. K., M. Stephens, and P. Donnelly. 2000. Inference of population structure using multilocus genotype data. Genetics 155:945–959.

Pritchard, J. K., X. Wen, and D. Falush. 2007. Documentation for STRUCTURE software: version 2.2.

Queller, D. C., and K. F. Goodnight. 1989. Estimating relatedness using genetic markers. Evolution (N. Y). 43:258–275.

Robinson, S. P., L. W. Simmons, and W. J. Kennington. 2013. Estimating relatedness and inbreeding using molecular markers and pedigrees: The effect of demographic history. Mol. Ecol. 22:5779–5792.

Schielzeth, H., and S. Nakagawa. 2013. rptR: Repeatability for Gaussian and non-Gaussian data. R package version 0.6.405/r52. http://R-Forge.R-project.org/projects/rptr/.

Van Oosterhout, C., W. F. Hutchinson, D. P. M. Wills, and P. Shipley. 2004. MICRO-CHECKER: Software for identifying and correcting genotyping errors in microsatellite data. Mol. Ecol. Notes 4:535–538.

Wang, J. 2007. Triadic IBD coefficients and applications to estimating pairwise relatedness. Genet. Res. 89:135–153.

Yu, H., and Q. Li. 2007. Development of EST-SSRs in the Mediterranean blue mussel, *Mytilus galloproviancialis*. Mol. Ecol. Notes 7:1308–1310.
